# Meteorosensitivity of patients with rheumatic musculoskeletal diseases

**DOI:** 10.1371/journal.pone.0333022

**Published:** 2025-10-06

**Authors:** Ulrich A. Walker, Katja Nettermann, Sebastian Walker, Adam Streeter, Veronika K. Jaeger

**Affiliations:** 1 Department of Rheumatology, University Hospital Basel, Basel, Switzerland; 2 Institute of Epidemiology and Social Medicine, University of Münster, Münster, Germany; 3 Hasso Plattner Institute, Potsdam, Germany; MVZ MED BAYERN OST Rheumatology, GERMANY

## Abstract

**Objectives:**

To evaluate if self-assessed rheumatic disease activity in patients with rheumatoid arthritis (RA), psoriatic arthritis (PsA) or ankylosing spondyloarthritis (AS) is linked to local weather.

**Methods:**

In this prospective, multicentre study in Switzerland, adult patients were asked to report geographic location and rheumatic disease activity by means of Routine Assessment of Patient Index Data 3 (RAPID3)) via a webApp. The associations between RAPID3 scores and weather data on the day of the RAPID3 entry and the preceding week was investigated through regression analysis.

**Results:**

238 patients were included (61.3% female). 100 patients had RA (baseline mean DAS28-CRP 2.8), 47 patients had PsA, and 91 patients had AS (mean BASDAI 3.8). 45.0% of all patients declared themselves as meteorosensitive. In the entire cohort, we found a weak negative correlation (r = −0.167) between RAPID3 and air pressure, the correlations between the remaining weather variables were weak (r < 0.1). In the subgroups of meteorosensitive AS and PsA patients, we identified negative correlations between RAPID3 and air pressure (r = −0.321 and −0.232 respectively). In the AS meteorosensitive group, there was also weak correlation between wind and RAPID3 (r = 0.144). In RA patients, only negligible correlations (r < 0.1) were observed, regardless of meteorosensitivity. In all analyses, also in the analyses of weather changes over the preceding week, we failed to identify effect sizes that exceed the minimal clinically important difference of the RAPID3.

**Conclusions:**

We did not find weather to be a major factor in disease activity of RA, AS and PsA patients receiving DMARD treatment.

## Introduction

Patient-reported outcomes (PROs) provide invaluable information on health, function, and quality of life (QoL) [1,2]. Clinical trials in rheumatoid arthritis (RA) have shown that the ability of PRO questionnaires to distinguish between active and control treatments is similar to that of laboratory tests and formal joint counts [3–5]. PROs have even been proven to be superior to physician-reported measures in determining the effects of disease-modifying anti-rheumatic drugs (DMARDs) on RA [5,6].

The Routine Assessment of Patient Index Data 3 (RAPID3) is a PRO tool for self-assessment of disease activity by the patient that captures three key domains: physical function, pain, and the patient global estimate of status. The RAPID3 takes about 1.5 minutes to complete, is reliable, valid and sensitive to change [6,7] Several studies have demonstrated tight correlations between RAPID-based self-assessments and objective clinical measures of disease activity in RA, psoriatic arthritis (PsA), and ankylosing spondylitis (AS) [7–13] In the Tight Control of Psoriatic Arthritis (TICOPA) trial for example, the correlation coefficient between the RAPID3 and the psoriatic arthritis disease activity score was 0.79 [12]. In an Indian study, the correlation coefficient between the RAPID3 and the DAS28-CRP in RA was 0.91 [13]. RA, PsA and AS share a similar disease burden and the use of the RAPID3 has been recommended in clinical practice [14–16]A patient who scores between 0–3.0 is defined as near remission; 3.1 to 6.0 as low severity; 6.1 to 12.0 as moderate severity; and above 12.0 as high severity [11].

RAPID3 scores were found to considerably fluctuate between clinical visits [11]. Many patients with rheumatic conditions report that changes in weather conditions aggravate their symptoms, raising the possibility that weather parameters could account for fluctuations in rheumatic activity [17,18]. Only a few studies, however, have evaluated the relationship between symptoms of rheumatic conditions and activity and weather [17–24]. Many different weather variables have been studied in the past, but most studies focussed on temperature, relative humidity and atmospheric pressure [18].

Given the good correlation of RAPID3 self-assessed rheumatic disease activity with objective disease activity measures and the ability to repeatedly and feasibly assess RAPID3 scores in regular time-intervals by means of smartphone technology, we aimed to reinvestigate the effects of weather on disease activity in a Swiss cohort of RA, PsA and AS patients.

## Methods

### Study population and design

This prospective, multicentre study was launched on the 15th of February 2016 and nested within the Swiss Clinical Quality Management (SCQM) registry, which prospectively follows patients with RA, PsA and AS. The SCQM registry was created in 1997 and health care providers in tertiary care settings and private rheumatology clinics participate in recruiting patients [25]. SCQM participants were offered to provide personal information by means of a webApp accessible by both, personal computers and mobile devices on Apple, Windows and Android systems. The webApp enabled patients to weekly self-assess their rheumatic disease activity by means of the RAPID3 score. Patients were also asked to provide their location at the time of their assessment in terms of postal codes. Patients were reminded weekly by SMS if they did not provide new data within two weeks. SMS reminders were cancelled if the patients did not respond to five consecutive weekly reminders. At the predetermined end of the study (14th of January 2021), the patients were also interrogated about whether or not they felt that their joint pain was influenced by weather conditions, e.g., if they identified themselves as meteorosensitive or not.

Meteorological data were provided by the Swiss national meteorological service (www.meteoschweiz.admin.ch) and consisted of pre-specified parameters such as hourly measured temperature, humidity, precipitation, hours of sunshine, pressure, and wind speed by individual weather stations. After study termination, the postal codes were manually linked to the closest Swiss weather stations based on the postal code provided by the participant. In case two or more weather stations were available at the same distance, the weather station with a similar elevation to the postal code was chosen. This procedure allowed to link the patients personal RAPID3 scores with the local meteorological data at the time of RAPID3 entry, and with meteorological measures in time windows preceding the RAPID3 entry.

### Statistical analysis

The association between weather and RAPID3 scores was explored using Tobit censored regression models to account for the predominance of zeros in the data, imposed by the lower limit of the scale. Robust standard errors were estimated to accommodate clustering of the data by patient. Analyses were adjusted by sex and a binary variable indicating whether or not patients had indicated any meteorosensitivity.

We first analysed the effects of seasonality in sex-adjusted Tobit regression models [26]. For seasonality, we used the meteorological definitions. Then, the RAPID3 was modelled in sex-adjusted Tobit regression models on each of the six weather variables: air pressure, temperature, wind speed, humidity, precipitation and sunshine. The weather observations used were those nearest to and preceding the time of entry of the RAPID3 score. In addition to the “on-the-day” observations, maximum changes measured in the corresponding weather variables over the preceding one-, two-, three- and seven-day periods were also modelled to assess the stability of the on-the-day observations and the influence of changeable weather conditions. For sunshine and precipitation, interest focussed on the average effects of these over the one, two, three and seven days preceding data entry for RAPID3. To adjust for variation in daylight affecting the total hours of sunshine, RAPID3 was also modelled on sunshine as a proportion of daylight over the previous 24 hours. In this exploratory study in which no correction for multiple statistical testing was applied, a 1% significance rate was applied to sift associations, after which the reporting of associations was based on effect sizes needed to translate into a 3 point RAPID3 change and interpreted cautiously. Attention was not only focused on the effect size, but also the consistency of any significant effects [27]. The clinical significance and effect size of the change variables on the RAPID3 were then compared with the minimal clinically important difference (MCID) for RAPID3 of 3-points [28]. The data was prepared in R Studio 4.3.1, statistical analyses were conducted using Stata version 13.

## Results

### Patient demographics

In total, there were 8607 RAPID3 observations on 238 patients, of whom 146 (61.3%) were female. We included 100 patients with RA (mean DAS28-CRP at baseline 2.8, SD 1.4), 47 patients with PsA (mean DAS-CRP at baseline 1.6, SD 1.0) and 91 patients with AS (mean BASDAI 3.8, SD 2.1). The mean age at the first data entry was 48 (SD = 13) years, ranging from 19 to 80 years and the mean age at diagnosis 39 years (SD = 12), ranging from 15 to 74 years. Further disease characteristics are provided in Table 1. There was a difference in the average RAPID3 score between men and women (p < 0.0001) with women having higher scores (8.1, SD 6.5) than men (7.1, SD 7.1). This sex difference was however not significant and weak when included in the model as an adjustment variable. RAPID3 scores did not correlate with disease duration in any of the three diseases under investigation (data not shown).

**Table 1:** Patient and disease characteristics.

	RA	PsA	AS
Patient number	100	47	91
Male – n (%)	26 (26.0)	27 (57.4)	39 (42.9)
Age in years at diagnosis – mean (SD)	42.0 (12.7)	38.9 (10.1)	36.1 (12.1)
Biological DMARD use %	28	30	35
JAK-inhibitor use %	14	0	0
Conventional synthetic DMARD use %	31	26	10
Apremilast use %	0	6	0
Median RAPID3 at study entry (IQR)	7.8 (8.1)	8.2 (8.3)	9.8 (12.2)
Median number of RAPID3 scores/ patient (IQR)	24.5 (73.5)	11 (39)	14 (49)
Median observation period in weeks (IQR)	106.6 (201.6)	48.6 (159.9)	49.4 (144.0)
Patients declaring themselves as meteorosensitive – n (%)	46 (46.0)	20 (42.6)	41 (45.1)

AS, ankylosing spondylitis; DMARD, disease-modifying anti-rheumatic drug; IQR, interquartile range; PsA, psoriatic arthritis; RA, rheumatoid arthritis; RAPID3, Routine Assessment of Patient Index Data 3 score.

### Weather variables during the observation period

A summary of the weather variables during the observation period is presented in Table 2. Many of the meteorological variables correlated significantly with each other, reflecting their expected interdependency (Table 3). For example, humidity had large negative correlations with temperature (−0.610) and sunshine (−0.631), while sunshine had the largest positive correlation with temperature (0.440). There was also a negative correlation between pressure and wind (−0.179).

**Table 2:** Summary statistics of weather measurements observed at patients’ locations at the time of app entry.

Weather variable	Units	N	Mean (SD)	Median (IQR)	Min	Max
Pressure	hPA	8321	966.3 (27.2)	973.0 (19.7)	803.6+	1005.7
Temperature	Centigrade	8403	12.3 (8.73)	11.9 (13.5)	−19.7	36.9
Precipitation	mm/h	8583	0.1 (0.7)	0 (0.7)	0	27.5
Relative humidity	%	8403	69.0 (19.3)	71.0 (32.0)	14.2	100
Sunshine	proportion of daylight	8285	0.3 (0.4)	0 (0.7)	0	1
Wind	km/h	8286	7.7 (5.8)	6.1 (6.1)	0	55.8

**Table 3:** Pearson correlation coefficients between meteorological variables at the time of RAPID3 scoring.

	Pressure	Temperature	Precipitation	Rel. humidity	Sunshine	Wind
Pressure	1					
Temperature	**0.136**	1				
Precipitation	**−0.040**	−0.021	1			
Relative humidity	0.001	**−0.610**	**0.153**	1		
Sunshine	−0.011	**0.440**	**−0.102**	**−0.631**	1	
Wind	**−0.179**	−0.001	**0.0539**	**−0.230**	**0.098**	1

Correlations in bold are statistically significant.

### Effect of meteorosensitivity and seasonality on RAPID3 scores

Approximately half of all patients (45.0%) declared themselves as meteorosensitive. The mean of each patient’s mean RAPID3 score was marginally higher in the meteorosensitive group (mean RAPID3 8.6) than in the weather insensitive group (mean RAPID3 8.2), but after adjusting for sex and clustered observations within patients, this difference was not significant (p-value = 0.40). Similarly, the prevalence of meteorosensitivity did not statistically differ between RA and PsA patients in remission (defined by a DAS < 2.6) and those not in remission (DAS > 2.6, p value 0.09 for RA. and 0.47 for PsA). There were also no statistical differences in remission (BASDAI <2) and non-remission (BASDAI ≥2) prevalences between meteorosensitive and insensitive AS patients(p = 0.26).

The RAPID3 varied between the meteorosensitive and weather insensitive groups according to the rheumatic disease (Table 4). In the RA group, the RAPID3 scores were comparable between the weather sensitive and the weather insensitive group, with the direction of the difference depending on the measurement of location (i.e., the mean and median), reflecting the skewness of the data. Among the AS and PsA patients, the mean and median RAPID3 scores were higher in the meteorosensitive groups. The differences between meteorosensitivity levels were significant only among the PsA patients, when modelling these using Tobit regression, adjusting for sex and clustering by patient (coefficient = 5.745, p-value = 0.003).

**Table 4:** RAPID3 scores by self-declared meteorosensitivity.

Meteorosensitivity	No	Yes
RA mean RAPID3 (SD) RA median RAPID3 (IQR)	7.1 (7.3) 4.7 (11.9)	6.1 (5.4) 5.3 (9.0)
PsA mean RAPID3 (SD) PsA median RAPID3 (IQR)	6.0 (4.9) 5.3 (8.5)	9.4 (6.9) 9.4 (13.0)
AS mean RAPID3 (SD) AS median RAPID3 (IQR)	8.5 (7.1) 6.5 (12.7)	10.3 (5.8) 10.7 (9.4)

When analysing the effect of seasonality on RAPID3 scores using Tobit models, there were no consistent or significant trends across seasons. The meteorosensitive patients in the AS group exhibited the largest deviation in RAPID3 scores across seasons, with scores in spring being significantly lower than the reference level of winter (p-value = 0.005). However, the difference was less than 1 point on the RAPID3 scale, and therefore not exceeding the minimal clinically important difference (MCID) for the RAPID3 [29].

In summary, we found higher RAPID3 scores in self-perceived meteorosensitive PsA and AS patients compared to weather insensitive patients but failed to detect a clinical meaningful relationship between disease activity and seasonality.

### Modelling RAPID3 on meteorological variables

We next investigated the relationship between weather parameters and RAPID3 at the time of documentation (Table 5). In the entire patient cohort, there was a weak negative correlation (r = −0.167) between RAPID3 scores and air pressure, whereas the correlations between all remaining weather variables were negligible (r < 0.1). Among individual disease subgroups, negligible correlations (r < 0.1) were also observed between all weather variables in the RA patients, regardless of their self-declared meteorosensitivity. In the subgroup of meteorosensitive AS patients, a negative correlation between RAPID3 and air pressure, was most pronounced (−0.321), followed by the RAPID3 and air pressure in the meteorosensitive PsA patients (−0.232). In the meteorosensitive AS group, there was also weak positive correlation between wind and RAPID3 scores (r = 0.144). In the weather insensitive AS patients pressure was also negatively correlated with RAPID3 (−0.199).

**Table 5:** Pearson correlation coefficients for weather variables and RAPID3 scores by weather sensitivity.

Disease group	RA		PsA		AS		Entire cohort
Meteorosensitivity	No	Yes	No	Yes	No	Yes	
**N patients**	54	46	27	20	50	41	238
Pressure	−0.020	**0.067**	−0.091	**−0.232**	**−0.199**	**−0.321**	**−0.167**
Temperature	0.024	0.002	−0.044	−0.053	−0.024	**−0.080**	−0.026
Precipitation	0.027	0.010	0.016	−0.029	**0.077**	−0.025	−0.023
Humidity	**−0.088**	0.000	0.003	0.022	0.032	**−0.072**	**−0.036**
Sunshine	**0.097**	0.002	0.026	0.011	0.007	0.027	**0.043**
Wind	**0.059**	**0.066**	0.041	0.085	0.021	**0.144**	**0.067**

Coefficients with p-values <0.01 are highlighted in bold.

We lastly analysed the association of the RAPID3 with changes of weather parameters for periods up to seven days prior to RAPID3 documentation (S1−S6 Tables). With regards to individual weather variables, the coefficients for on-the-day pressure were significant with p-values <0.01 for the AS and PsA patients, with coefficients of −0.0388 and −0.0987 respectively (S1 Table). Once stratified by weather sensitivity, then the results were only significant for the weather sensitive group. For AS patients, the pressure difference required to account for a 3-point change in RAPID3 scores was 75 hPA and in PsA, the coefficients indicated that pressure at the time of RAPID3 entry would have to be 30 hPA lower to account for a 3-point change in RAPID3 scores. During the whole observation period, the maximal absolute pressure change that was recorded within 24 hours was however lower (29.6 hPA). The coefficient for the effect of wind observed at the time of app entry in the PsA group was also significant and remained significant with the inclusion of change variables in the model (S2 Table). Although a positive correlation between wind speeds on the day of measurement and RAPID3 could be observed in all disease groups, this relationship was strongest in the AS group and in the meteorosensitive PsA group. When stratified by meteorosensitivity, then the effects were only apparent in weather-sensitive patients in the AS and PsA groups and of all disease groups combined, with the highest coefficient of 0.143 in the weather-sensitive PsA group implying a meaningful 3-point drop in RAPID3 could be achieved with approximately 21 km/h lower average wind speeds. During the whole observation period, the maximum change in wind speed over 24 hours was 44.7 km/h.

There were no correlations with temperature, humidity and precipitation changes (1 day change, 2 day change, 3 day change and 1 week change) that would indicate a clinically meaningful 3-point change in RAPID3 among the entire cohort as well as in the disease subgroups, neither for meteorosensitive, nor for insensitive patients (S3–S5 Tables).

When analysing the entire cohort, RAPID3 was found to negatively correlate with the absolute hours of sunshine/day or the sunshine measured as a percent of daylight counted up to 7 days before app entry in patients (S6–S7 Tables). Negative coefficients indicated an inverse relationship in all disease groups, i.e., lower disease activity with more sunshine hours. However, with the largest coefficient of for sunshine in the day before app entry for all participants being −0.044, a meaningful 3 point reduction in RAPID3 would not be achievable in the preceding 24 hours. There was also a significant effect of sunshine accumulated over the previous week in the PsA patients, although with a coefficient of −0.023, a patient in this group would have to experience on average 18.6 hours of sunshine a day over the preceding week to affect a 3 point drop in RAPID3.

In summary, we found significant correlations between RAPID3 and pressure or wind changes in the meteorosensitive AS and PsA subgroups of patients, but the differences required to trigger a clinically meaningful change in RAPID3 would be related to unrealistic weather extremes.

## Discussion

In this study, smartphone technology was employed to assess the influence of weather on rheumatic disease activity. This App-based approach has demonstrated feasibility, as there was a high degree of engagement with the technology in terms of regular data entry by patients. Prior assessment had shown that patients rated the tool as easy and intuitive-to-use [11]. Also, elderly patients and patients with no prior smartphone experience were able to use the webApp; hence this webApp-based approach is generalizable to a wide range of patients [11]. A prior study has demonstrated a good correlation (r = 0.63) between RA activity as assessed by physician by means of the DAS-28 and patient-rated disease activity in terms of RAPID3 scoring with this webApp [11]. The correlation between the RAPID3 and the BASDAI in AS patients was found to be 0.75 in another study [30].

One strength of our investigation is that we were able to compare patients identifying themselves as meteorosensitive with those who do not. Across all rheumatic disease subgroups almost half of the Swiss patients identified themselves as meteorosensitive. When comparing RAPID3 scores between meteorosensitive and insensitive patient groups, the meteorosensitive AS and PsA patients had higher RAPID3 scores. The exact causes of meteorosensitivity are poorly understood. Psychological factors, such as depression, also play a significant role in the perception of weather-related symptom changes. For example, the depression scores were found to significantly contribute to symptom severity in RA patients, suggesting that psychological state can modulate the perception of pain and discomfort [31].

When analysing the correlations between individual weather parameters and RAPID3 scores, we found inverse correlations with air pressure in the whether sensitive AS and PsA patients. Further analyses found consistent but week correlations with air pressure and wind. Since pressure correlates negatively with wind in our dataset and since wind speed is related to pressure differences, the identified wind findings are possibly secondary to pressure differences. The magnitude of changes required to translate into a clinically meaningful change of disease activity is however not expected to occur outside of weather extremes. In line with these findings, we also failed to detect clinically meaningful effects of seasonality on disease activity.

Our findings are in line with studies identifying small and only clinically insignificant effect sizes of weather parameters on RA patient pain [21,22,23] Interestingly, two of the studies [20,21] suggested that in RA patients, increasing pain was associated with higher barometric pressure, where as our study and the results from one other group [32] pointed rather into the opposite direction.

Although our study examined a larger patient sample than previous studies [19–24] and compared the effects of weather on a variety of different inflammatory rheumatic diseases it has also limitations. The results of this purely Swiss study must also not be generalized to other ethnicities, and geographical or meteorological peculiarities in other countries. The RAPID3 has also not been correlated with enthesitis indices in AS and PsA. We were therefore unable to draw conclusions about the contribution of individual disease manifestations in the axial spondyloarthritis that we investigated. We also cannot exclude the possibility that a larger effect of weather parameters on disease activity was blunted by the benefits of treatment, as most patients received DMARDs and consequently had only low disease activity. It must also be kept in mind, that despite the good correlations of the RAPID3 with disease activity, RAPID3 responses may be influenced by emotional factors like mood or stress, comorbidities and musculoskeletal pain from non-arthritic origin. We were therefore for example unable to fully separate the effects of rheumatic disease activity and underlying osteoarthritis on RAPID3 scores and the self-assessment of meteorosensitivity [33].

Our findings do not support the anecdotal beliefs about a directional relationship between rheumatic disease activity and weather, similar to previous observations in fibromyalgia [34]. The effect size calculations show that the relationships that we detected are probably of little clinical relevance. The discrepancy between our finding and common belief may originate in part from a confirmatory bias [34]. Alternatively, selective matching may be a possible explanation [22]. Selective matching leads people to look for changes in the weather when they experience increased pain, whereas they pay little attention to the weather when their pain is stable [22].

In summary our study suggests that the effects of weather conditions on the symptoms of RA, PsA and AS are minimal and highly variable, even in patients declaring themselves as meteorosensitive. Therefore, weather is not a major factor in disease management.

## Supporting information

S1 Table Coefficients from Tobit modelling of RAPID3 on the nearest pressure observation, measured at or just before the time of RAPID3 score entry adjusted for sex and in models adjusting for the maximum fluctuation over the preceding one, two, three and seven days. Abbreviation of ATDE: at time of data entry.
(TIF)

S2 Table Coefficients from Tobit modelling of RAPID3 on the nearest wind speed observation, measured at or just before the time of RAPID3 score entry adjusted for sex and in models adjusting for the maximum fluctuation over the preceding one, two, three and seven days. Abbreviation of ATDE: at time of data entry.
(TIF)

S3 Table Coefficients from Tobit modelling of RAPID3 on the nearest temperature observation, measured at or just before the time of RAPID3 score entry adjusted for sex and in models adjusting for the maximum fluctuation over the preceding one, two, three and seven days. Abbreviation of ATDE: at time of data entry.
(TIF)

S4 Table Coefficients from Tobit modelling of RAPID3 on the nearest relative humidity observation, measured at or just before the time of RAPID3 score entry adjusted for sex and in models adjusting for the maximum fluctuation over the preceding one, two, three and seven days. Abbreviation of ATDE: at time of data entry.
(TIF)

S5 Table Coefficients from Tobit modelling of RAPID3 on cumulative precipitation measured during the preceding 1, 2 and 3 days, and 1 week. (TIF)

S6 Table Coefficients from Tobit modelling of RAPID3 on cumulative sunshine measured during the preceding 1, 2 and 3 days, and 1 week. (TIF)

S7 Table Coefficients from Tobit modelling of RAPID3 on sunshine measured as a percent of daylight over the previous 24 hours to adjust for seasonal variation in daylight. (TIF)
