# Supplementary figures and images for: Meteorosensitivity of patients with rheumatic musculoskeletal diseases
Source: PLoS One. 2025 Oct 6;20(10):e0333022. doi: 10.1371/journal.pone.0333022 (PMC12500097; doi:10.1371/journal.pone.0333022)

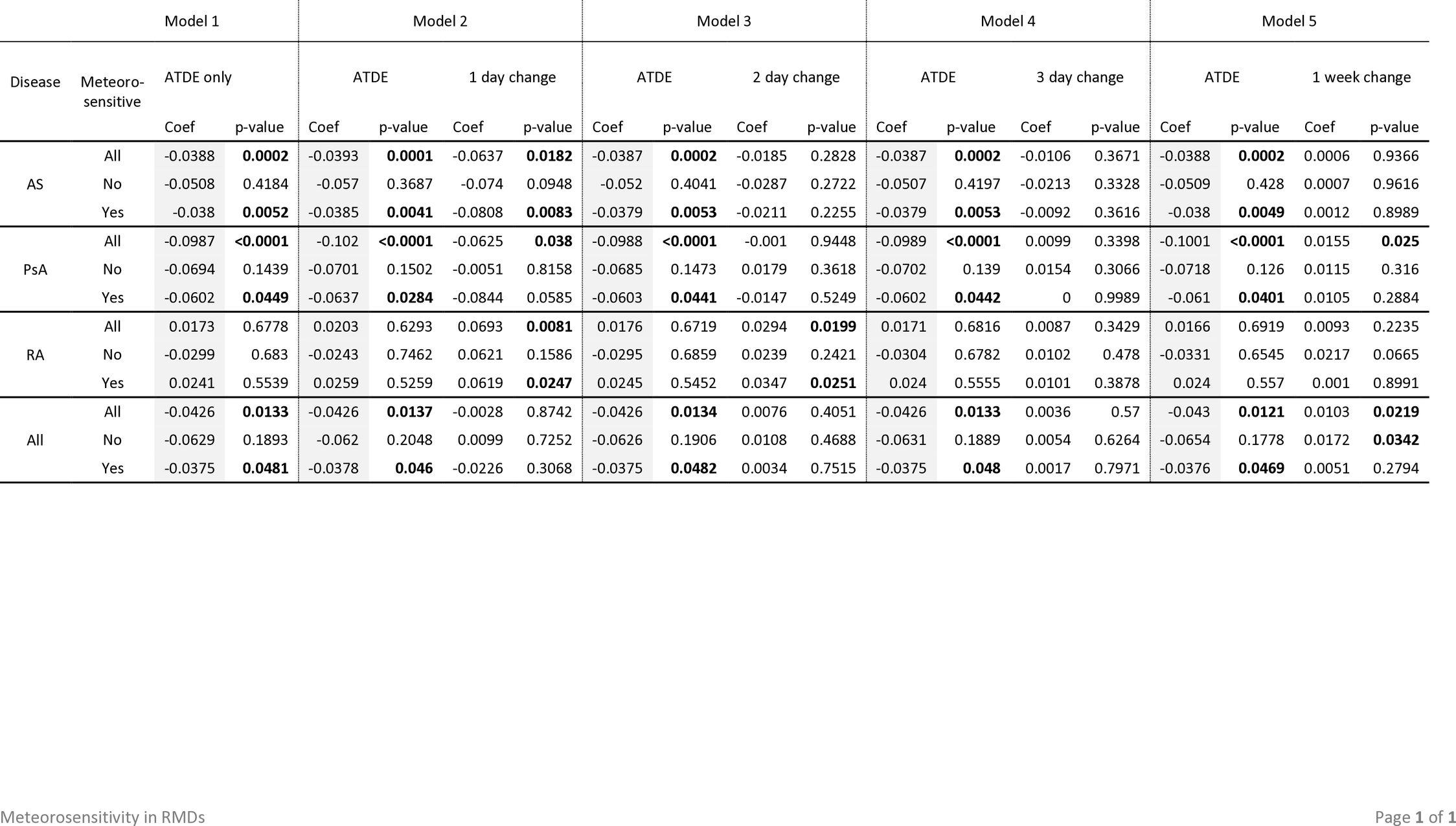

Supplement: S1 Table — Abbreviation of ATDE: at time of data entry. (TIF) [file pone.0333022.s001.tif]

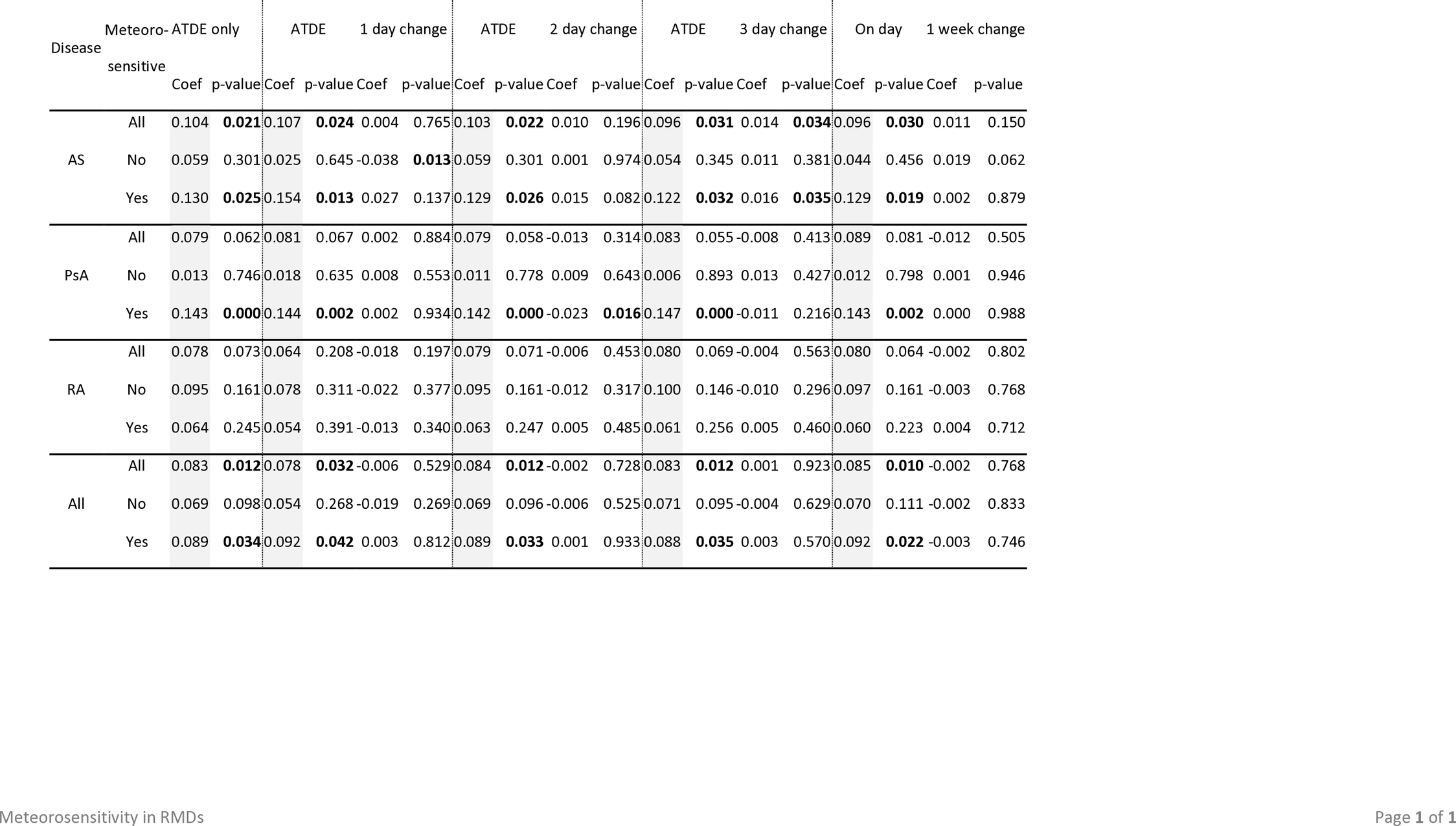

Supplement: S2 Table — Abbreviation of ATDE: at time of data entry. (TIF) [file pone.0333022.s002.tif]

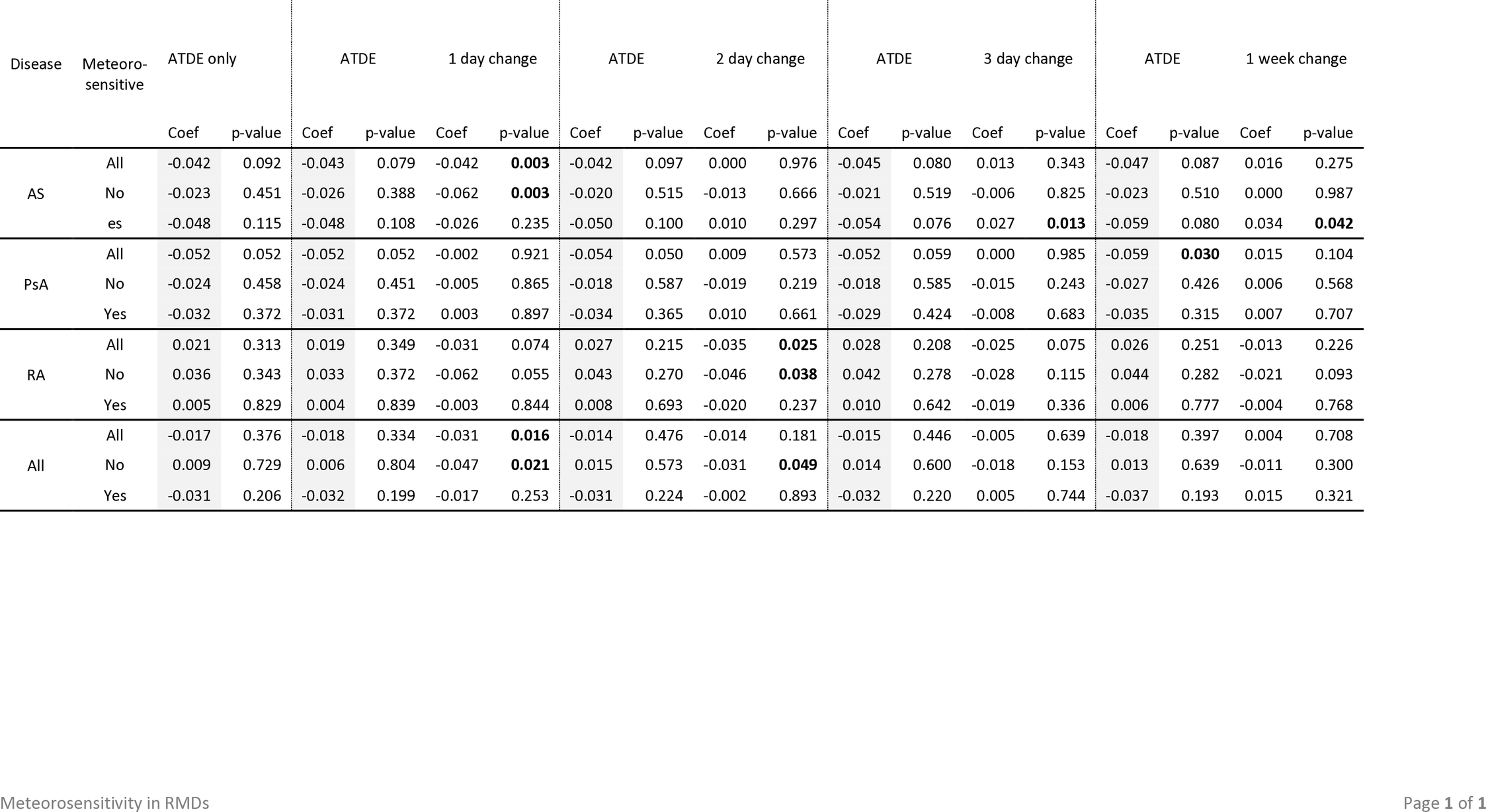

Supplement: S3 Table — Abbreviation of ATDE: at time of data entry. (TIF) [file pone.0333022.s003.tif]

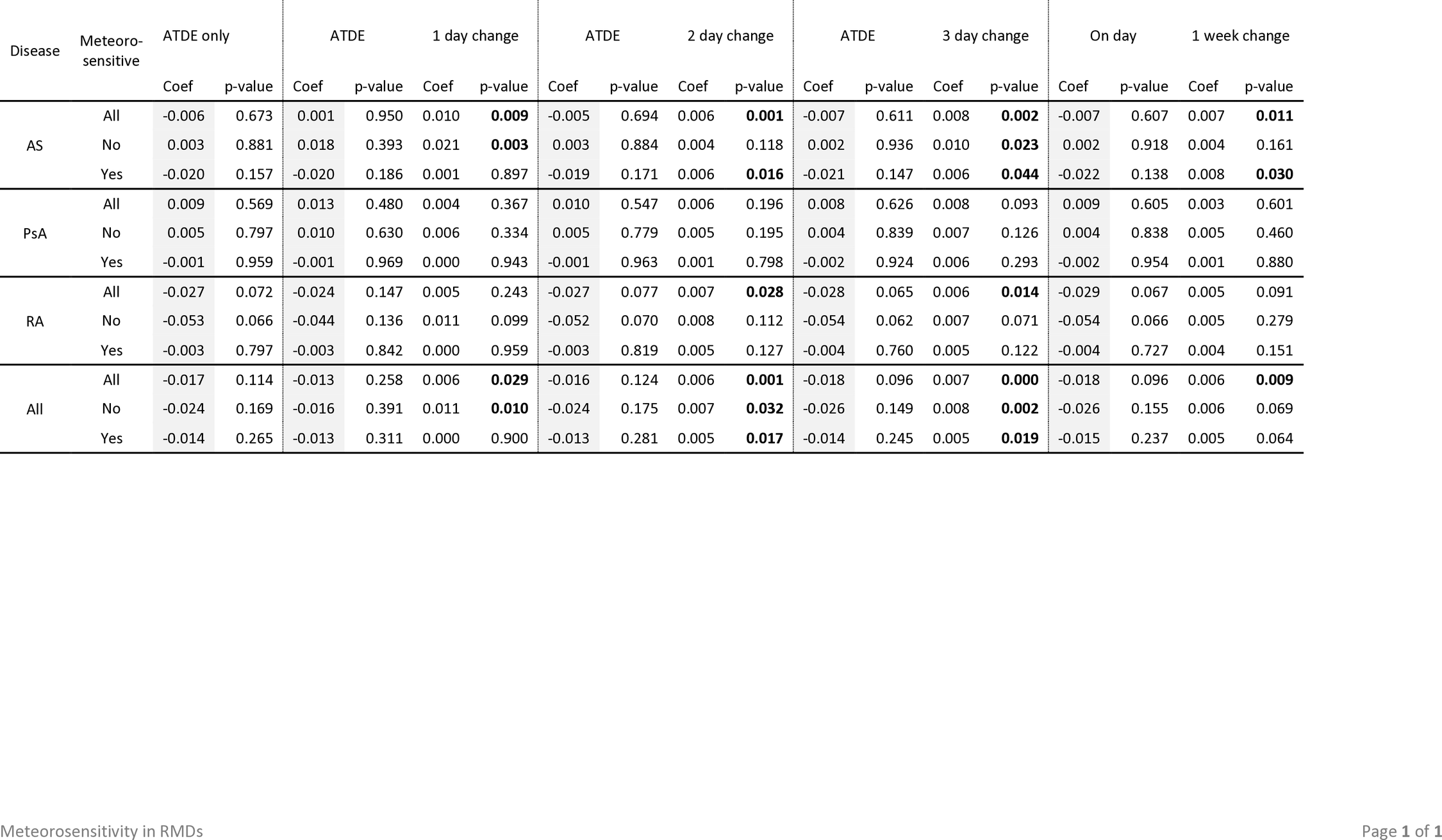

Supplement: S4 Table — Abbreviation of ATDE: at time of data entry. (TIF) [file pone.0333022.s004.tif]

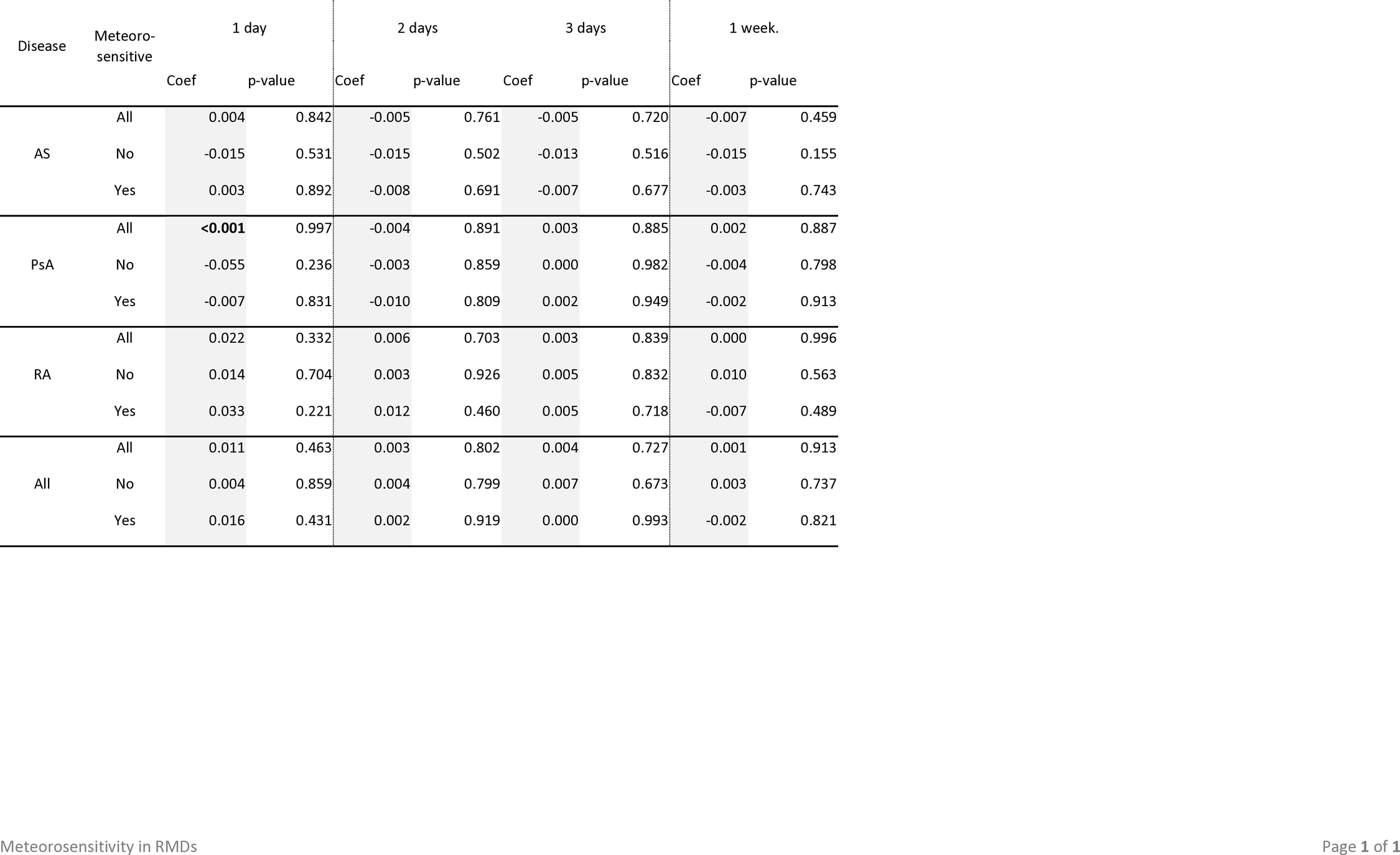

Supplement: S5 Table — (TIF) [file pone.0333022.s005.tif]

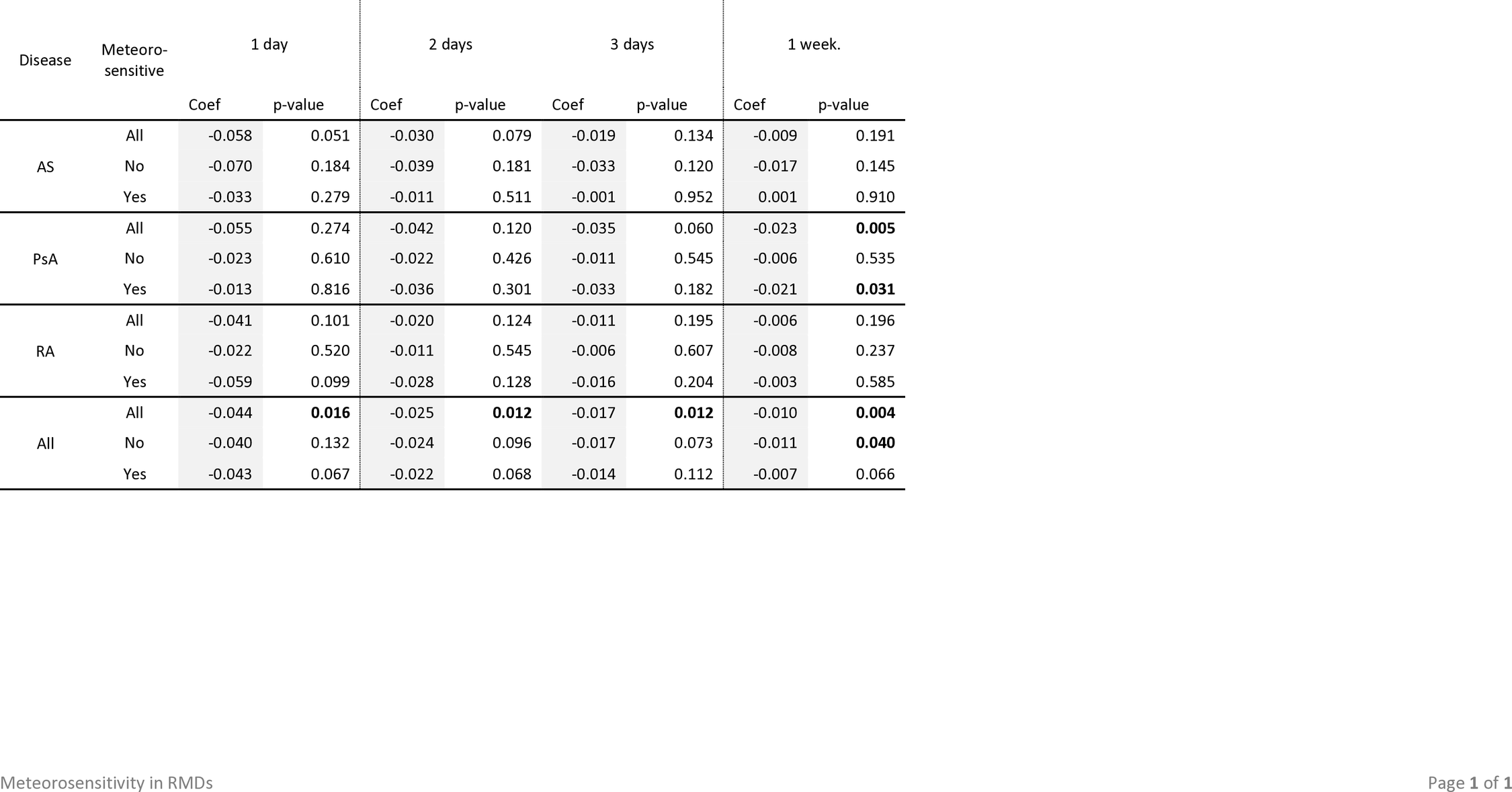

Supplement: S6 Table — (TIF) [file pone.0333022.s006.tif]

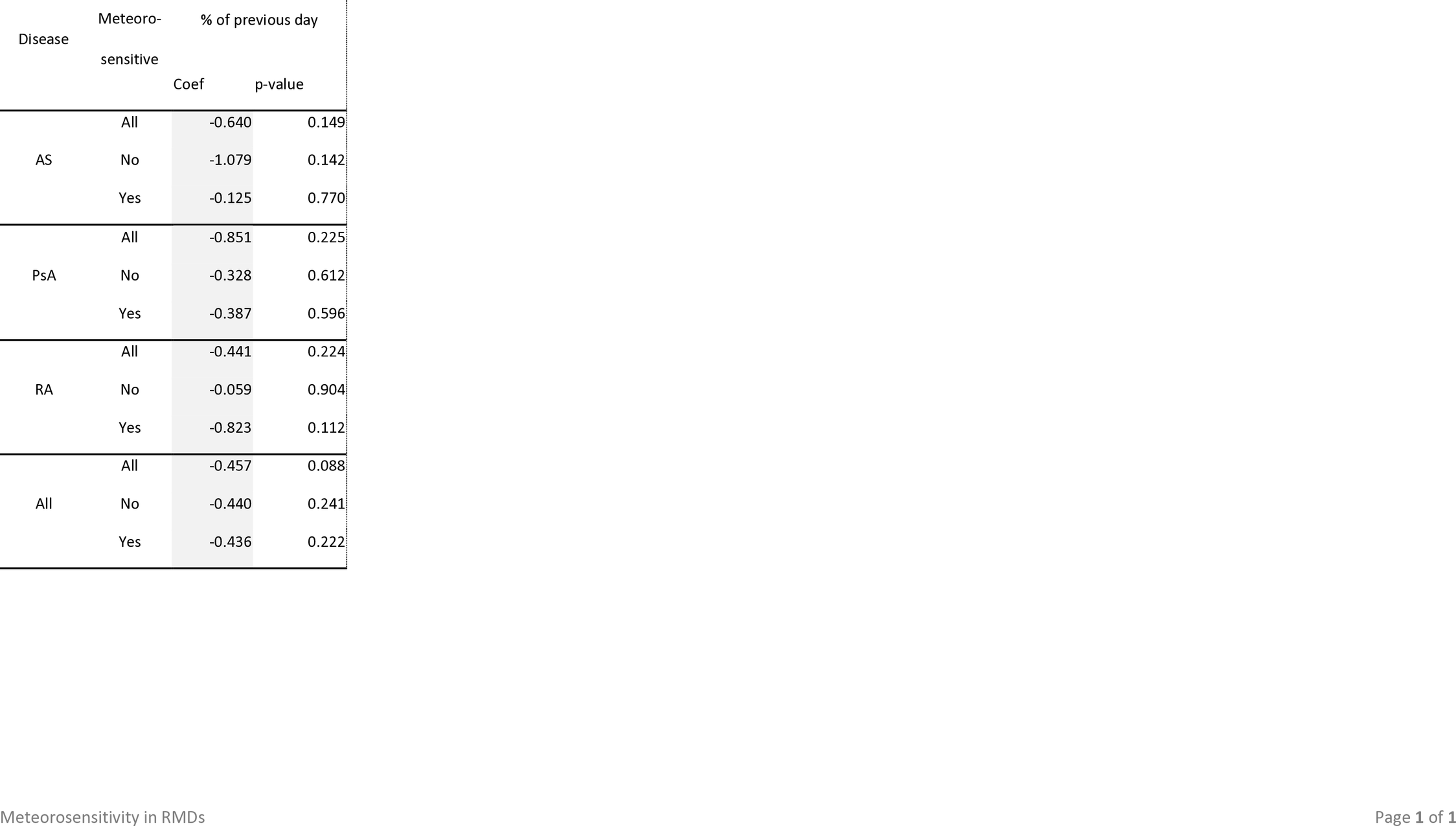

Supplement: S7 Table — (TIF) [file pone.0333022.s007.tif]
